# On a handshake: business-to-business trust in the biosecurity behaviours of the UK live plant trade

**DOI:** 10.1007/s10530-023-03054-y

**Published:** 2023-04-04

**Authors:** Chris R. J. Pollard, Mariella Marzano

**Affiliations:** Forest Research, Northern Research Station, Roslin, EH25 9SY UK

**Keywords:** *Xylella fastidiosa*, Biosecurity, Trust, Decision-making, Plant trade

## Abstract

The movement of plants through the ornamental plant trade presents a major source of risk for the introduction and spread of plant pests and pathogens. To minimise the likelihood of infested or infected plants moving through the value chain, individual businesses can adopt a range of biosecurity practices to prevent introduction on site, as well as detecting and then containing or eradicating any plant pests or pathogens present. However, a major additional source of risk is the arrival of unhealthy plants sourced from a supplier. Using the example of bacterial plant pathogen *Xylella fastidiosa* which has a large host range and potentially devastating economic and environmental impacts, we highlight the importance of trust when businesses navigate the risks of sourcing plants. Through interviews and a survey with a range of plant businesses, we show (i) how two general types of risk—relational risk associated with suppliers acting in good faith, and performance risk associated with suppliers having the ability to perform as expected—can be applied to the challenge of sourcing healthy plants, (ii) how businesses respond to these risks through behaviours based on trust and control, and (iii) the potential outcomes of trust-based and control-based behaviours in the presence of a hard to detect pathogen such as *Xylella fastidiosa*. We conclude that trust is a significant component in decision-making in the live plant trade, and as such any behavioural interventions designed to encourage better biosecurity practices in the industry should capitalise on this understanding to strengthen responses and avoid undermining of effort.

## Introduction

The movement of live plants for the purposes of trade present a major risk of introducing and spreading plant pests and pathogens into and around the UK (Chapman et al. 2017; Marzano et al. 2017; Spence et al. 2020). Trade has been directly implicated in the introduction of pests and pathogens such as *Phytophthora ramorum* (Brasier 2008), ash dieback (caused by *Hymenoscyphus fraxineus*), and oak processionary moth, *Thaumetopoea processionea,* (Marzano et al. 2020), and Asian longhorn beetle, *Anoplophora glabripennis* (Porth et al. 2015). Pests and pathogens have a broad range of impacts, including damage to ecosystems and incur financial costs; the impact of ash dieback alone is projected to cost the UK £15 billion due to safety felling, replanting, and loss of ecosystem services (Hill et al. 2019). Whilst dealing with existing pests and pathogens impacting on UK treescapes, decision-makers also need to prepare for, or mitigate against, new and emerging threats.

*Xylella fastidiosa* (Xf) is a vector-borne bacterial pathogen which to date, has not been found in the UK. Xf was first described over 100 years ago in the Americas and has now been detected in at least 36 countries globally (https://gd.eppo.int/taxon/XYLEFA/distribution). It has a range of host plant species numbering in the hundreds (European Commission 2018) and is the causal agent of severe diseases such as Pierce’s Disease of grapevines, plum leaf scald, citrus variegated chlorosis, oak leaf scorch, and olive quick decline syndrome (Hopkins and Purcell 2002; Saponari et al. 2019). Host species relevant to the UK include: lavender, *Lavandula spp.*; cherry and plum, *Prunus spp.;* rosemary, *Rosmarinus officinalis*; grapevine, *Vitis vinifera*; and sycamore, *Acer pseudoplatanus*, but there are many more. The first identification of Xf in Europe was in the Italian olive groves of Puglia in 2013, which by 2017 had spread across 53,500 ha to over 6.5 million olive trees (Scortichini 2020). At the time of writing, it has also been detected in France, Spain, and Portugal (https://gd.eppo.int/taxon/XYLEFA/distribution). Infected host plants can be asymptomatic, or show symptoms shared by other pathogens, making detection without laboratory testing difficult. Xf is spread between plants via xylem sap feeding insects (*Hemiptera, Auchenorryncha*) (Rapicavoli et al. 2018). However, it is the import of infected live plants for planting (rather than movement of the vector insect) which is seen as the most likely entry route for Xf into the UK, with an outbreak potentially resulting in considerable negative ecological, social, and financial impacts (Defra 2020). In naive environments such as the UK where Xf is not yet found, there is much uncertainty surrounding how human-mediated processes such as the import of infected plants would impact the eco-epidemiology of Xf, alongside a range of other interacting factors: pathogen biology; host biology; pathogen/host interactions; insect vector biology; and abiotic conditions (Occhibove et al. 2020). In this article we explore current biosecurity practices of stakeholders that trade in two known Xf hosts—Lavender and Cherry—with specific attention paid to value chain relationships. We aim to derive new understanding of the business to business (B2B) relationships between suppliers and customers in the live plant trade, concentrating on the risks faced by businesses when relying on suppliers to meet biosecurity expectations.

## What actions can a business take to reduce the risk of spreading pests?

**Fig. 1:**
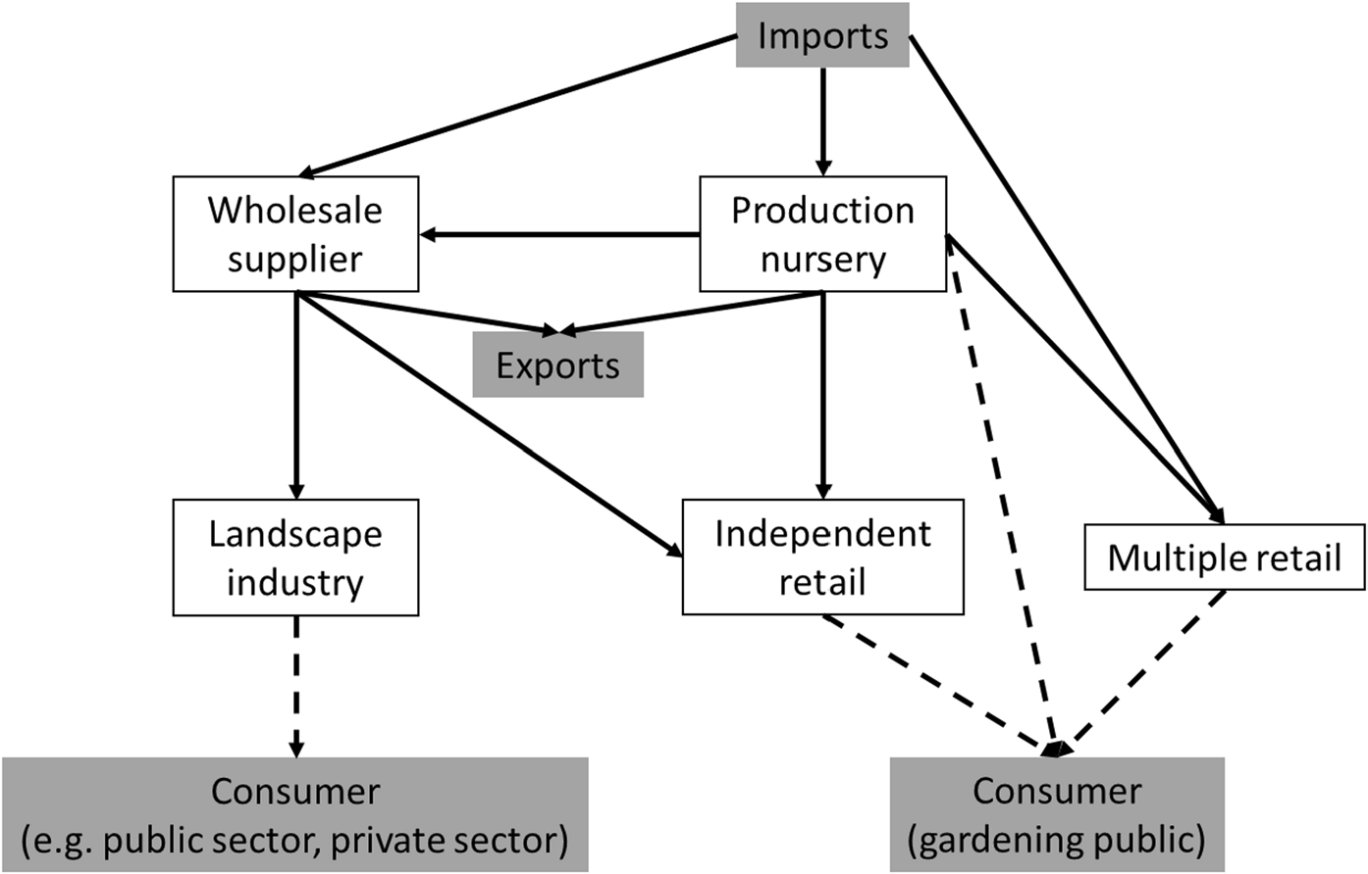
Potential flow of plants through a simple live plant value chain. Solid lines indicate business-to-business (B2B) movement of plants, dashed lines business-to-consumer movement of plants. Simplified version of figure from Hulme et al., (2018)

The behaviours of people and businesses involved in moving plants into and around the UK will impact the potential for the spread of any pests and pathogens attached to those plants. Identifying key decisions, and the underlying reasons for them, is an important step towards encouraging better biosecurity behaviours. Uncertainty in human-mediated factors arises from the varied stakeholder types associated with each stage of an outbreak (Dandy et al. 2017). Key amongst these types are businesses in the live plant value chain such as growers, nurseries, wholesalers, retailers, and landscape professionals, whose behaviours will mediate the containment or spread of infested or infected plants. A live plant value chain is made up of a minimum of two actors but likely many more, between which plants are traded (Drew et al. 2010) (Fig. 1). Outbreaks in a plant trade network are directed, with the disease being passed from an infected business to susceptible businesses, the latter of which then becomes infected and can pass on the pest or pathogen to others (Pautasso and Jeger 2014). Likelihood of transmission through trade will be dependent on the volume of plants moved from infected to susceptible actors, which will generally be dominated by a downstream flow from growers to nurseries to wholesalers to retailers to consumers (Fig. 1). However, movement of plants and equipment (e.g. pallets and trucks) will also occur upstream, sideways, and bidirectionally resulting in the risk of pest and pathogen transmission from multiple types of interaction. In order to prevent the flow of pests and pathogens along a value chain, an individual or business should minimise the chance of: i) a pest or pathogen entering their business from infected actors; ii) a plant becoming infected on site; and iii) a pest or pathogen leaving the business to potentially infect other susceptible businesses.

What constitutes good onsite biosecurity practices will depend on the type of business (nursery, retailer, landscape contractor etc.) but actions for protection against pests and pathogens include: quarantining new plant arrivals; using a contained composting or incineration facility; and disinfection of boots, tools, vehicles, and other equipment. Dunn and Marzano (2019) found differences in the actions taken between UK nurseries and garden centres with for example, boot washing stations more prevalent in garden centres than nurseries (> 45% and < 20%, respectively), and tool/pot disinfection stations more prevalent in nurseries than garden centres (> 50% and < 15%, respectively). When asked to give reasons why they did not undertake biosecurity actions, nurseries and garden centres most commonly responded that they did not believe that specific action was appropriate for their business, rather than having too great a financial or time cost (Dunn and Marzano 2019). UK plant businesses have suggested decreasing bureaucracy and increasing customer demand as potentially successful incentives to adoption of more biosecurity behaviours (Green et al. 2021), potentially through use of an accreditation scheme (Marzano et al. 2021). These data imply a high perceived level of control at their own sites, i.e. businesses do not have to *rely on others* to manage their own onsite biosecurity. This is similarly the case when plants are dispatched to other businesses, where checking of plants for signs of pest or pathogen prior to release is within the control of the dispatching business.

## How can businesses manage biosecurity risks involving their suppliers?

A business is not likely to have a high level of control when dealing with incoming plants from its suppliers where the onsite biosecurity practices and processes of the supplier will impact the level of risk present for the business receiving the plants. For clarity, we will hereafter refer to the actor receiving the plants and making the risk-based decision as the ‘business,’ and the actor supplying or dispatching the plants as the ‘supplier.’ When sourcing plants from a supplier, a business faces a degree of risk that the supplier will pass on a pest or pathogen, with the ability to undertake biosecurity processes such as quarantining or individual plant checks under pressure to process incoming plants (e.g. for sale) as quickly as possible. Uncertainty surrounding the magnitude of that risk can be reduced by checking the supplier’s biosecurity awareness and practices, and the risk itself reduced by requiring the supplier to increase the number and extent of their biosecurity actions. It could be assumed that all other characteristics equal, no business would choose a less biosecure supplier over a more biosecure one. At the very least, a business may be ambivalent or unaware about biosecurity. Certainty of suppliers’ biosecurity practices is less likely attainable by a business, with rigorous scrutiny of suppliers’ biosecurity practices potentially costly. A business therefore faces a biosecurity risk when selecting their live plant supplier. When making sourcing decisions they may consider this perceived risk alongside other supplier characteristics such as product price, stock range, stock volume, delivery speed, and supply reliability.

## Risk and trust

A strategic alliance between two partner businesses is formed when both parties can gain more from cooperation than if acting individually and where interactions are governed by relationships rather than purely by market forces (Kostis and Näsholm 2019; Rinehart et al. 2004). Examples include co-development of a product, sharing of resources, or some supplier-customer relationships. Cooperation carries an element of risk for each party that the other will not fulfil expectations, whether on purpose (also termed ‘defection’) or not.

Research into B2B value chain relationships has led to a wide range of categorisations and frameworks for highlighting the importance of and attempting to understand the role of trust in decision-making. In a review of research on B2B relationships, Jamaluddin and Saibani (2021) identify trust-based as one of three common types of relationship (the others being power-based, and a mixture of cooperation and competition). Mayer et al. (1995), define trust in an organisational setting as being distinct from similar concepts such as cooperation, confidence, or predictability, due to factors of risk, choice, and lack of control. Trust can be dependent on perceptions of ability, integrity, and benevolence, whilst also affected to a lesser or greater degree by the personal propensity of a potential trustor and the wider situational context (Fulmer and Gelfand 2012; Mayer et al. 1995). Alternatively, shared values, good communication, and not behaving opportunistically are significant predictors of trust in value chains (Mlaker Kac et al. 2016). Schoorman et al. (2007) highlight the importance of but not requirement for, reciprocity between organisations who have trust-based relationships, and that emotion and mood may affect perceptions of trustworthiness. Trust can also be described as a rational behavioural choice or as more complex set of interpersonal relationships (Lewicki et al. 2006). Stern and Coleman (2015), describe the components of trust between actors in social-ecological systems as: dispositional (relating to the individual and their personal history and perceptions of norms); rational (based on calculated assessments or prior performance); affinitive (based on emotional judgments); and procedural (relating to control systems which increase predictability of behaviours). Trust in another organisation can be located at both an individual scale (employees of a business) or at team or organisational scale where there may be no consensus of trust from those individuals of a team, but the team does display trust in an external actor (Fulmer and Gelfand 2012).

**Fig. 2:**
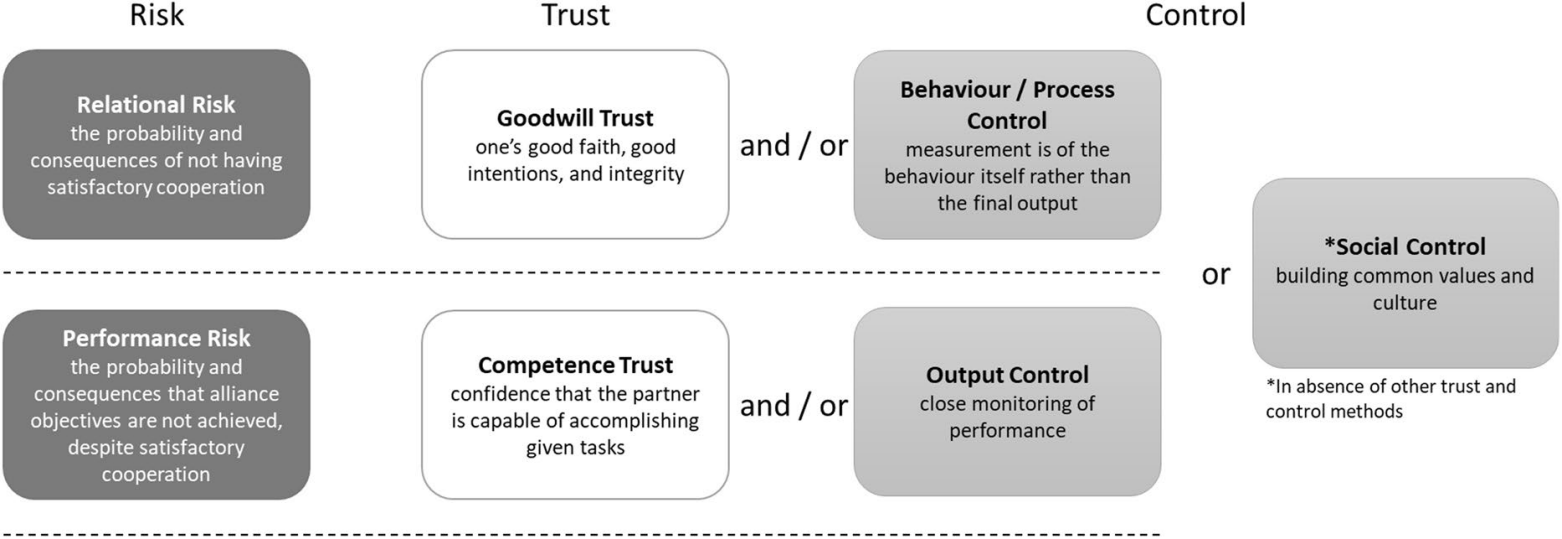
Summary of risk types, and appropriate trust or control strategies. Adapted from Das & Teng (2001)

To explore B2B relationships in the UK plant trade we use a framework of risk and trust from Das and Teng (2001, 2004), which identifies types of risks faced by independent businesses collaborating, and the subsequent methods of managing that risk: trust and control. The risk-based view of trust (Das & Teng 2001, 2004) can be used to examine how cooperating businesses respond to risk by means of trust-based or control behaviours. The framework sees trust as an expectation of how the other party will behave in a risky B2B situation. There are then two categories of risk (performance and relational) each with a corresponding type of trust (competence and goodwill, respectively) and control (output and process controls, respectively). A third type of control, social control, is included where other types of trust and control fall short (Fig. 2).

Relational risk comes from the possibility that a partner will not fulfil expectations because they see an alternative course of action which is more beneficial to themselves (Das & Teng 2001, 2004). A business faces lower perceived risk when they have goodwill trust; where the partner is trusted to act in good faith without the need for extra rules, checks, or monitoring. Alternatively, where goodwill trust is lower, businesses can reduce relational risk by employing behaviour controls (also termed ‘process controls’ in the original framework), which involves scrutiny of the systems and processes required for a partner to complete tasks as expected. Under behavioural controls, a business is seeking to ensure that the workings of their partner are compatible with their own values. The second type of risk, performance risk, comes from the possibility that the partner will not fulfil expectations because they do not have the capacity, ability, or skills required, even if they are trying their best. This risk is perceived as lower in the presence of competence trust, where the partner is trusted to deliver the product as expected. In cases of lower competence trust, output control can be used, which involves tight monitoring of the product, giving prompt and detailed feedback where the product is not delivered satisfactorily. Output control seeks to raise the attention of a partner to product deficiencies and prompt them to increase their capacity, ability, or skills to meet the objectives. A final type of control is useful in circumstances where the other types of trust and control have not been effective. Social control relies on actions to build a foundation of common values and culture between partners, the lack of which can be the cause of the other trust / control ineffectiveness. In situations where failure to deliver on the part of one partner is because of small or large value chain issues (e.g. breakdown of delivery vehicle, third party supplier going out of business) or wider system shocks (e.g. currency movements, change in international trade agreements), this may not affect trust at all depending on the second partner’s perception of the issue or shock itself. However, it could be perceived by the second partner as a failure of competence, for example a lack of preparation for rare events.

The risk-based view of trust allows analysis of interview data using risk-trust-control categories, to better understand the role of trust in business-supplier relationships, whether it is stated overtly as a driver of supply decisions or not. The movement of plants and, therefore, the potential movement of pests and pathogens between suppliers and customers is regulated at least partially by the sourcing decisions made by customers. A successful biosecurity relationship would be typified by both supplier and customer being free from pests and pathogens as much as possible, with swift and effective corrective actions taken in the case of detection or outbreak.

Using qualitative and quantitative data from interviews and surveys we focus on the stated importance of risk and trust in decision-making and explore how businesses in the UK live plant trade choose plant suppliers to work with, the role played by biosecurity in that decision-making, and how those decisions could impact biosecurity at the business and sector scales. By determining how characteristics of risk and trust are associated with biosecurity behaviours, we highlight potential behavioural risks to plant health and identify broad interventions for encouraging behaviours which could protect and enhance plant and tree health. Our research offers an expanded view of the importance of human behaviour associated with the commercial movement of plants into and around the UK focussing on decision-making in the absence of specific regulators of supplier behaviour such as trading laws, or adherence to voluntary certification or labelling schemes. We discuss the potential impacts such behaviour may have on biological invasions and plant pest and pathogen events.

## Methods

This research was conducted as part of the Brigit Project (https://www.jic.ac.uk/brigit/), which aimed to increase the UK’s capability to prevent establishment of vector-borne plant pathogens, specifically *Xylella fastidiosa* and to increase preparedness to respond should it be introduced. Social research data was collected using semi-structured interviews and a questionnaire of individuals in the UK trade of live lavender plants and sweet cherry trees; plants deemed to be of high risk for importation of Xf. Focus species were selected in consultation with Brigit project pathology and policy partners.

### Semi-structured interviews

The authors conducted interviews between July 2019 and September 2020, lasting an average of 1 h. One interview was conducted by video call, the remainder by phone. Interviews were conducted remotely for both convenience of the participant and because of restrictions in place due to the COIVD-19 pandemic. Where phone lines were of poor quality, participants were called back immediately, or at a later date. Stakeholder groups of interest consisted of the following types: grower; garden centre; nursery; wholesaler; landscape contractor; landscape architect/garden designer; and consultant. Participants were recruited using three methods. The first method involved a long-list of potential participants created using publicly available information on businesses (companieshouse.gov.uk). Categories of business included in the first step are shown in Table 1. The order of the long list was then randomised and starting at the top of the list, internet searches were made to identify if the business fit the criteria as a group of interest and if so, any publicly available contact details were collected and added to the entry. Businesses which did not fit the criteria or did not have contact details were excluded from the list. Contact was then made by email or phone call. For the second method, businesses which were named as suppliers or customers during interviews were noted and permission requested to contact them for a potential interview. The third method employed a more targeted approached for recruitment to increase coverage not achieved by methods one and two alone. This involved internet searches for businesses in the stakeholder groups of interest specifically located in Wales, Northern Ireland, and Scotland, and for growers of lavender and cherry in the UK as well as recommendations from Brigit team members and partners.

**Table 1:** Categories of UK business included in download from Companies House (http://download.companieshouse.gov.uk/en_output.html) as potential participants

Growing of citrus fruits
Growing of other non-perennial crops
Growing of other tree and bush fruits and nuts
Growing of pome fruits and stone fruits
Growing of vegetables and melons, roots and tubers
Landscape service activities
Logging
Mixed farming
Plant propagation
Retail sale of flowers, plants, seeds, fertilisers, pet animals and pet food in specialised stores
Silviculture and other forestry activities
Support services to forestry
Urban planning and landscape architectural activities
Wholesale of flowers and plants

The interview schedule included questions around (i) supplier choice, and (ii) relationships with suppliers and customers, with a focus on (but not limited to) biosecurity behaviours. Interviews were recorded and transcribed verbatim by transcription companies. Transcripts were checked and corrected where possible by CP using original audio files. Interview transcripts were coded by CP using NVivo (QSR International). Analysis of coded data was conducted by CP and MM. Coding was based around the interview schedule structure, with additional nodes to code for types of risk, trust, and control as defined by Das and Teng (2001, 2004). Forty-four semi-structured interviews were conducted with UK-based businesses in the live plant trade (Table 2).

**Table 2:** Interview number and code by business type, size and case study species

		Number of interviewees (*n* = 44)	Interviewee codes
Business type			
	Garden centre	8	GC1-GC8
	Nursery	9	Nu1-Nu9
	Grower	5	Gr1-Gr5
	Wholesaler	5	Wh1-Wh5
	Wholesaler/nursery	1	WN1
	Landscape contractor	12	LC1-LC12
	Landscape architect/garden designer	3	LA1-LA3
	Other	1	Ot1
Business size			
	Micro (1–10 employees)	22	
	Small (11–50 employees)	11	
	Medium (51–250 employees)	6	
	Large (250 + employees)	5	
Case study species			
	Lavender	19	
	Cherry tree	22	
	Neither	3	

### Questionnaire

Participants were recruited and surveyed by phone by a contract research company (Latimer Appleby Ltd) in March 2021. Criteria for recruitment included being a trader of lavender and or sweet cherry and located in either one of the counties of East Sussex or West Sussex, in the south of England, UK. Recruitment was restricted to the Sussexes as they had been identified by Brigit epidemiological modellers as high risk for the introduction of *Xylella fastidiosa* in the UK (Occhibove & White, per comms.) and to minimise the volume of searching required by recruiters.

The questionnaire collected data on business type, size, and trade; Xf and its impacts; and plant health practices. All questionnaire participants were based in East or West Sussex, UK and self-identified as one of grower (10%), nursery (48%), wholesaler (15%), or garden centre (27%). The number of plants sold annually varied widely within and between business types from 2500 to 2.8 million.

Of those surveyed, 59% bought in lavender or cherry trees from suppliers. These 59 businesses were asked the relationship duration of their three biggest suppliers, with the majority of relationships lasting for 1–5 years (31%) or 6–10 years (39%). 17% of businesses had supplier relationships of 11 years or more. Businesses used a range of suppliers to provide their required plants, as very few suppliers (7%) accounted for more than 75% of the total stock brought into surveyed businesses.

Responses were inductively coded by CP and MM resulting in 15 categories of response (see Table 3). Categories were not mutually exclusive. Categories were then grouped into three higher-level categories according to the risk-based view of trust: Competence trust; Goodwill trust; and Other (neither Goodwill nor Competence Trust) by Das and Teng (2001, 2004). Due to data collection method (no elaboration on the question) there was likely overlap between some initial categories, thus the higher-level categories are more reliable for interpretation. Where there was ambiguity as to whether initial categories could be goodwill or competence trust (e.g. share similar attitude could be either), the ‘other’ higher-level category was assigned. Data management, and descriptive and test statistics, were carried out by CP in R version 4.0.2 (R Core Team 2020).

**Table 3:** Categorisation of survey responses—What makes a supplier trustworthy?

Initial categorisation	Higher-level categorisation
Honesty	Goodwill trust
Relationship building	Goodwill trust
Shows willingness	Goodwill trust
Will refund/replace stock	Goodwill trust
Communicates well	Goodwill trust
Has transparency	Goodwill trust
Has clear processes	Competence trust
Has good knowledge	Competence trust
Has good products	Competence trust
Don’t know/just have a gut feeling/instinct	Other
Good reputation	Other
Treat their staff well	Other
Share similar attitude to our business	Other
They have longevity	Other

## Results

### Risk, trust, and control

We initially explored relationships between businesses and their former and current suppliers and found that interviewees were assessing risks based on trust and mitigating those risks as well as, if required, with control actions. When describing what separates a good supplier from a bad supplier, one interviewee summarised: “*Plant quality. Customer service*.” [GC6]. Poor performance from these two named areas; low plant quality and bad customer service, mirror the two categories of risk, Performance Risk and Relational Risk, respectively (Das & Teng 2001).

When describing characteristics of successful supplier relationships, several interviewees used the word ‘trust’ unprompted. For example, interviewees highlighted: “*we just stick to the same growers that basically we trust”* [Gr2], and “*it’s more important to have a relationship with your supplier and to trust them, that would be the crucial thing for me”* [LC6].

### Risk

Risks described by participants were coded into relational and performance risks; suppliers not acting in good faith versus not being able to meet the standard required, respectively. Or more simply, relational risk comes from deliberate decisions (e.g. mislabelling, not paying due compensation, shipping substandard plants, not conducting agreed biosecurity checks), and performance risk comes from accidental failures (e.g. not recognising a pest during checks, lack of awareness of emerging plant health threats).

#### Relational risk—the probability and consequences of not having satisfactory cooperation

Interviewees often indicated a concern that one or a small number of actors could have a large biosecurity impact, as just one diseased plant could cause a serious outbreak. The perception that a rare conscious transgression of good biosecurity practice could undo the hard work of the vast majority of well-meaning businesses was illustrated by one nursery owner, who showed frustration that their hard work could come to nothing if others did not also engage in good biosecurity practice: “*it doesn’t matter if 99.9% of the nurseries in the country follow what we do it only needs one […] the damage is done, it’s too late and we can do what we want but what’s the point?”* [Nu3].

Risk can be attenuated with the use of legal contracts, but they tend to be rare in the UK horticulture sector especially amongst smaller businesses, according to one micro-business interviewee: “*ornamental horticulture supply industry is bereft of specific contracts which surprised me […] I couldn’t believe that 99% of horticultural commitment is a handshake”* [Wh5].

Businesses perceived that their suppliers could knowingly take a chance on biosecurity, hoping that they won’t be the one to facilitate a pest or pathogen event. Interviewees said that for untrustworthy suppliers “*the risk element isn’t important to them”* [Nu4], and that even with knowledge or warning “*It doesn’t stop them doing it”* [Nu5].

Interviewees cited the reasons for such poor behaviours could include maximising of profits (although sometimes just to make ends meet), the ability to sell quickly/reduce administration, and a lack of care or respect for the horticultural industry. One interviewee described cutting corners themselves, sourcing olive trees from a supplier about which they knew very little, so an order could be fulfilled in time.

Interviewees listed further concerns about potential poor supplier behaviours including deliberate mislabelling of plants by suppliers to avoid species restrictions, selling or shipping plants which are known to be infected with a non-notifiable disease, not paying compensation for plants sold which have been shown to be diseased, withholding information about a high failure rate for a particular product line and, poor communication on the projected outcomes of contract growing.

#### Performance risk—the probability and consequences that alliance objectives are not achieved, despite satisfactory cooperation

Interviewees also highlighted various risks surrounding the ability of suppliers to deliver the desired health standard of plant products. Knowledge and awareness of plant pests and pathogens was the main noted area of performance risk, with the potential for infested or infected plants to move through the value chain not because of corner-cutting or malice, but because surveillance expertise was lacking. Interviewees talked about knowledge and awareness along the length of the value chain: “*[plants are] probably handled four times before they come to me. Are all those people being taught about the problems?”* [GC2] and sourcing plants from regions with different pests and pathogens: “*the further you move away from the origin of the plant, [people] further down the chain aren’t going to be as familiar with that particular pest or disease, are they?”* [LA2].

Lack of awareness was particularly thought to be the case for instances of uncommon pests and pathogens of which a supplier would inevitably have less experience. For this reason, the risk of not identifying an infested or infected plant before dispatching it to a customer could remain even if suppliers were highly competent in the administrative biosecurity processes required in the trade, such as plant passporting and certification. One interviewee commented: “*you can have all the traceability and plant passports etc.* *all working perfectly fine, but if you haven't got that instinct on the ground with a pair of eyes and that knows what they’re looking for, that’s where it’s going to fall down”* [LC2]. However, there was a general feeling that checks and records processes such as plant passports were beneficial to biosecurity of the sector.

There can be a struggle to keep pace with new pest and pathogen threats and with changing or updated biosecurity advice. One interviewee described one of their suppliers: “*the owners I think may be quite elderly now and there’s just a culture there of not really moving fast enough with the times and not maybe thinking that it’s necessary to be more organised and hygienic”* [LC5]. Several interviewees highlighted non-specialist traders such as supermarkets as potentially high-risk actors, perceiving a lack of the expertise amongst shop staff needed to identify infested or infected plants.

Interviewees also expressed concern that they themselves could suffer from a lack of knowledge around identification of plant pests and pathogens, thus increasing performance risk for their own customers. This is a key tenet of performance risk: those responsible for the risk are trying to do their best.

### Trust

#### Goodwill trust—one’s good faith, good intentions, and integrity

The key method to reduce perceived relational risks is through development of goodwill trust. Interviewees described relationships with suppliers which show behavioural characteristics emanating from goodwill trust, how this trust was developed over the time of the relationship, and the positive impacts of being in such a relationship. The data collection methods used did not explicitly identify the motivations of an interviewee’s supplier, but instead concentrated on the perception held by the interviewee of their supplier(s), as described by the interviewee. In the case of goodwill trust, this perception would likely include an implicit evaluation by the interviewee of their supplier’s motivations towards them.

One aspect of goodwill trust in suppliers was described by interviewees as having a personal relationship with an individual who knew what they wanted and would see that only the plants suitable for them were dispatched. One typical comment was “*he doesn’t send the rubbish [plants] and wouldn’t tolerate rubbish himself”* [LC6].

If there was a problem with plants, goodwill trust was evident where a business knew that the supplier would refund quickly and easily, and that communication to solve the problem would be well-received. One interviewee said “*I sent them an email and within 24 hours, they had a credit note back to me and I just dumped those* *[plants], it was no problem”* [GC4]. Another described how their supplier could have acted in an opportunistic, self-interested way on an occasion of supplying substandard plants, but they had chosen not to: “*they were able to agree to replace those trees free of charge, but I know they could have argued that it was an act of God, it was beyond their control”* [LC11].

Suppliers taking accountability for substandard plants (for example by offering no-argument refunds), sometimes resulted in a business behaving reciprocally, themselves avoiding opportunistic actions, “*we would ask our supplier to bear the brunt of much of that cost. We do sometimes go halves on it*” [Wh4]. Business behaviour such as this rewards the supplier, encourages them to continue acting in the interests of their customer, and builds trust between both parties.

#### Competence trust—confidence that the partner is capable of accomplishing given tasks

Mitigation of perceived performance risks associated with suppliers comes from competence trust, in the supplier’s ability to complete tasks as expected. Interviewees who displayed competence trust in their suppliers described how their expectations of the plant products delivered were met.

A typical description of the behaviours of a competence-trusted supplier was provided by one interviewee: “*they’re very cost-efficient, their prices are fairly good and the plants are good as well […] they’re fairly hassle free, as it were, I don’t have to go to a nursery, […] the order is fulfilled and so far I haven’t had any problems with them”* [LC5]. Plants sourced from these suppliers are described as good quality and delivered on time to the required specifications including size or variety/species. What makes a plant ‘good quality’ was not specifically discussed during interviews, but a healthy-looking plant and a plant which grows well were both descriptors of quality which can result from an absence of pest or pathogen (although not always). One garden centre owner described how plants growing well are a measure of quality, without direct knowledge of the plant being entirely disease free: “*[customers say] “Your plants are wonderful, I come to you because any plants I’ve bought from you have grown” and that’s good and I’m pleased to hear that. And, it gives me confidence that I am selling the best possible quality or the highest quality plants possible without hopefully any pests and disease”* [GC4].

Trust in a suppliers’ competence to deliver the required product meant conducting checks of supplier processes and practices were perceived as unnecessary. The cost of conducting such checks was described as prohibitive, or at least inefficient to perform, given time and finance pressures on independent businesses. However, almost all those interviewed would visually check plants on arrival from suppliers, with the most common method of dealing with substandard plants being either disposal or returning them to the supplier.

Competence trust appeared to be built in two ways: firstly, personal experience of good quality plants being supplied over time, with interviewees describing that they were satisfied to stay with a supplier as long as this continues; secondly, new suppliers being ‘tested’ for example by submitting an order to one or more suppliers and evaluating the supplier on their competency to deliver the required plants.

### Control

#### Behavioural control—measurement is of the behaviour itself rather than the final output

Where relational risk is perceived as a problem, and goodwill trust lacking, businesses can help mitigate against potential issues by first taking steps to understand and assess the relational risk, then where required taking behavioural control actions themselves. Generally, behavioural control actions involve examining the various processes of how a supplier operates and trying to influence the supplier to modify these processes if they are unsatisfactory.

In the UK plant trade, behavioural control usually begins with a representative of the business visiting supplier sites to assess plant husbandry and site cleanliness. Interviewees variously described what they check when visiting a supplier; one thorough example is presented here “*you go to a nursery, you check it for cleanliness, the paperwork, its turnover of stock, who it supplies, you drill into that supplier to understand what market they’re supplying, are they buying it in from Italy the day before, putting it into the ground and then selling it out, is it their own production, is it a one hit wonder, [then if they are a good supplier you] can you use that nursery year after year after year”* [Nu7]. Performance during such assessments is a proxy for suppliers and customers sharing and being willing to invest in maintaining the same values (for example in cleanliness).

Using formal measures of process assessment, such as standardised checklists or scoring systems was rare, with businesses preferring to rely on their own personal experience and expertise to judge suppliers. One interviewee abandoned using a formal scoring system because they felt that it could sour relations with the supplier: “*We used to try and score it, but I think at the level that we were doing it at, it almost came across as patronising”* [Wh5]. In this case the business had attempted a behavioural control, but to maintain the relationship they had to endure some relational risk until they built enough trust with the supplier over time. The same interviewee also noted that “*the industry has been, I suppose, small enough that trust and experience is worth a lot to those of us that work in [the industry]”* [Wh5], illustrating they felt that due to repeated interactions between a limited number of actors in the industry, opportunism was kept at bay.

The act of checking processes is a control as it signals to the supplier what is important to the business. Interviewees also gave examples of asking for processes to be upgraded, with the threat of taking their business elsewhere a consequence of supplier inaction: “*we’ve actually recommended certain processes [….] we can’t actually dictate to the suppliers; what we can do is actually say that if it doesn’t improve then we will be changing our source of stock and that usually works”* [GC1].

#### Output control—measurement of the final output, rather than preceding behaviours

Where performance risk was seen as a problem, and competence trust was lacking, businesses could help mitigate against potential issues by careful monitoring of the plants delivered and reporting back to their supplier when dissatisfied. The resulting feedback would aim to spur suppliers to change their practices and meet product expectations of the receiving business. A typical description of behaviour that can be categorised as output control was that “*you send the plants back and they soon get wise that you won't accept anything* *[substandard]”* [GC5].

Suppliers showing good customer service (building Goodwill Trust, see above section) may offer to refund or replace unsatisfactory plants. Output control differs as businesses seek to increase the quality of future outputs by highlighting issues with the current outputs. Interviewees described their processes as “*isolate and report”* [GC1] or more specifically “*we take photographs of [substandard plants] if they’ve come off the van, send them and then they go in the skip”* [GC2].

Some interviewees saw the desired changes in the plant product, but more commonly either no change was seen, and the business changed supplier, or the business was satisfied with being refunded. The latter behaviour did not reduce the risk of being supplied substandard plants again but did reduce the risk of negative financial consequences of a substandard supply.

On occasion, failed attempts at output control would result in the business staying with a particular supplier but not for the affected product, which they would purchase elsewhere. One example given was regarding box blight, a fungal infection of *Buxus* spp. of shrubs, where a grower provided infected plants. The business concerned switched supplier for *Buxus* spp. but kept using the supplier for other plants. Here, substandard biosecurity did not tarnish the supplier enough that other products were questioned, and competence trust in the supplier was maintained.

### What makes a supplier ‘trustworthy’?

To expand our understanding of characteristics which represent a trustworthy supplier, we conducted a survey asking plant traders who bought in live plants (*n* = 59) to name up to three things which make a supplier trustworthy. Responses were inductively coded into 15 different categories, which were then ultimately grouped into three higher-level categories according to the risk-based view of trust: Competence trust; Goodwill trust; and Other (could not be categorised distinctly as either Goodwill nor Competence Trust). Examples of categorised responses are shown in Table 4. Of 104 responses by 59 respondents, 54 (51.9%) were coded as Goodwill Trust; 15 (14.4%) were coded as Competence Trust; and 35 (33.7%) were coded as Other.

**Table 4:** Example responses from plant traders when asked what make a supplier trustworthy. Responses coded using the risk-based view of trust

Higher-level category	Example coded responses
Competence Trust	In depth plant knowledge
	Clear guidelines in place for staff on how to handle plants
	Clear uncomplicated answer
	Supply what you need
	Good range of good quality stock
Goodwill Trust	Good and fair returns policy
	Being on your side
	Willingness to help
	Open, honest
	Sharing responsibility
Other	The depth of their client list
	The way their staff look. Are they happy
	Longevity
	You just know—I can't explain it any more than that
	Somebody who works the same way we do in our own business

## Discussion

How a plant pest or pathogen spreads is dependent on the combination of characteristics of the pest or pathogen itself, the host species, and (as is the case with *Xf*) any vector species required for transmission. In such a human-controlled system as the live plant trade, human behaviour is the fourth key component influencing the potential of pest and pathogen spread (Kleczkowski et al. 2019; Occhibove et al. 2020). Thus, how a business addresses perceived risks of using a supplier (through trust or control) will interact with the characteristics of a plant pest or pathogen, the host, and vectors to mediate disease spread.

Business-2-business trust has been defined in many ways (e.g. Lewicki et al. 2006; Das & Teng 2004). A third of our survey respondents, themselves described traits of trustworthiness which we could not confidently code as either distinctly competence or goodwill trust using the risk-based view of trust. Given the self-reported importance interview respondents placed on trust as a driver for their decision-making, there could potentially be a significant proportion of plant businesses making decisions based on their own definition of trust which is not fully captured with the framework we have used. Such a result illustrates the complexity of trust and the measurement of trust, and suggests clear room for other research lenses and further exploration into perceptions of trust by actors in plant trade B2B relationships.

Still, our application of the risk-based view of trust, is useful as a lens to better understand decision-making in the UK plant trade by illustrating the differences in behaviour which can and do appear depending on the perceived type of risk faced in a supplier-customer relationship. Relational risk (RR) and performance risk (PR) in the live plant trade are related to the expectations held by one party about behaviours of another. These could be an expectation that the plants supplied look attractive, an expectation that a supplier will refund for faulty goods delivered, or an expectation that a supplier is knowledgeable about their product and so will offer accurate advice. Biosecurity risk (BR, the risks caused by plant pests and pathogens) can be associated with a subset of these expectations, for example the expectation that plants delivered are disease-free, or that value chain cleanliness follows agreed standards. But BR can also be present even when expectations are fulfilled, and the assessment of RR and PR is low, as is the case with pathogens such as *Xf* which have asymptomatic or cryptic characteristics.

**Fig. 3:**
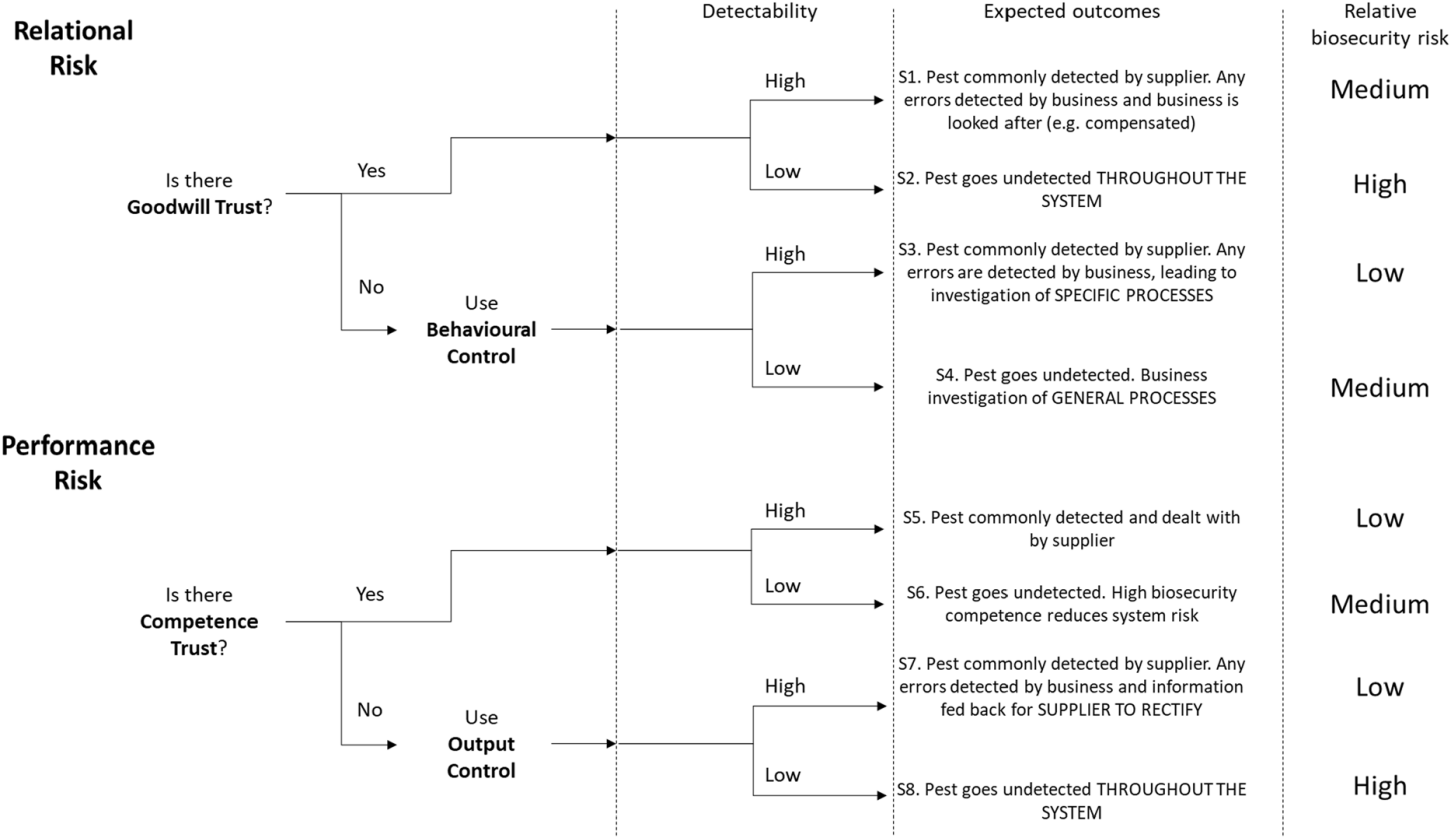
Potential outcomes and relative biosecurity risk based on the combination of perceived risk, trust, control, and pest/pathogen detectability. Pest or pathogen is abbreviated to ‘pest’

Creating scenarios which differently combine types of risk, trust, and control with likelihood of disease detection allows insight into (i) how actions taken in response to risk can have different outcomes depending on the pest or pathogen characteristics; and (ii) ways in which interventions designed to change or respond to behaviours may be less or more effective in reducing the spread of pests and pathogens. Figure 3 shows a flow chart of eight potential situational outcomes and relative biosecurity risk (low, medium, high), based on the combination of perceived risk type (RR, PR), trust type (goodwill trust, GT; competence trust, CT), control type (behavioural control, BC; output control, OC) and pathogen detectability (low, high). Scenarios here are not exhaustive, with outcomes describing how a business could act based on the risk-based view of trust, for example by investigating supplier processes in response to detection of a pest or disease. Should a business not be satisfied with supplier behaviour and also not be able to influence change, then their options are limited to tolerating increased risk, or terminating the partnership and seeking a new supplier.

*LOW biosecurity risk scenarios*—three scenarios (Fig. 3) rated low BR indicate where the pest or pathogen (hereafter abbreviated to pest) is easily detectable. Where relational risk is tackled by behavioural control (S3), a pest would commonly be detected by the supplier, with any errors leading instead to detection by the customer who would enact behavioural controls to investigate the specific processes leading to the error. Where performance risk is perceived as low due to the presence of competence trust (S5) the pest would be detected and dealt with by the competent supplier. Where output control is used due to a lack of competence trust, (S7) the pest would still be commonly detected by the supplier but in the case of errors, the customer would detect the pest and feedback the data to the supplier leaving the supplier themselves to rectify the clear errors and increase their competence in this area.

*MEDIUM biosecurity risk scenarios*—one scenario of easy detection and two of difficult detection are rated as medium biosecurity risks (Fig. 3). With perceived relational risk low due to the presence of goodwill trust, an easily detectable pest (S1) should be detected by the supplier with any errors detected by the customer who is looked after in good faith, for example by issuing a refund and replacing affected plants. In this scenario, the pest is prevented from spreading further, but the issues causing the presence of the pest may not be addressed as both supplier and customer feel the goodwill actions are a satisfactory response—the pest may appear again but both parties know that the commercial relationship will remain intact. Where the pest is not easily detectable (as with *Xf*) and behavioural control is used to tackle relational risks (S4), the pest may go undetected by both supplier and customer (Fig. 3). However, the investigation of processes and systems taken by a customer in the name of behavioural control aim to raise the level of general (rather than specific to one pest) biosecurity in the supplier, offering some protection against a less detectable pest. Similarly, where competence trust is present and thus performance risk is perceived as low (S6), there is already a higher level of biosecurity competence which should offer protection against the occurrence of a pest even if a pest is less likely to be detected.

*HIGH biosecurity risk scenarios*—two scenarios of low detectability are rated as high relative biosecurity risk (Fig. 3). Where perceived relational risk is low due to goodwill trust (S2), the relationship between supplier and customer is characterised by behaviours where identified unhealthy plants, are compensated for. Here there is no pressure from the customer towards the supplier to increase general or pest-specific biosecurity practices as they are automatically compensated. Detection of pests will stop further spread but not reoccurrence, thus low detectability will result in both spread and reoccurrence. Where performance risk is tackled using output control (S8), the simple feedback mechanism from customer to supplier which reports substandard plants and drives future healthy plant supply does not function as neither the customer nor supplier detect the pest. Again, pests will spread and reoccur.

### Behavioural interventions

Building better biosecurity in the live plant trade most keenly concerns the behaviour of those in the industry themselves as potential ‘human vectors’ of plant pests and pathogens (Dandy et al. 2017). However, encouragement of behaviours such as keeping premises clean, quarantining new arrivals, avoiding risky species or sources, and training staff to recognise and report pests and disease, concerns a wider range of stakeholders affected by the impacts of plant pests and pathogens including government, environmental NGOs, and end users (plant buying public or businesses) (Dandy et al. 2017). Plant biosecurity can be approached as a public goods issue, and methods for successful collective management can be suggested from wider common pool management design principles based on an agreed goal, defining boundaries of responsibility, development of rules and norms, self-governance, graduated sanctions, shared action, and a strong working relationship (Bagavathiannan et al. 2019; Ostrom et al. 1999). Reducing the impact of pests and pathogens which can be carried asymptomatically or showing only cryptic symptoms can be tackled with testing or development of resistance plants. However, such interventions would be expected to carry costs both of development and implementation and bring with them issues of who should bear those costs. Our analysis shows how trust between businesses is a cornerstone of decision-making in the UK industry and, therefore, attention should be paid to utilising existing trust to enhance collective biosecurity action. We have highlighted where, in medium and high-risk scenarios, there are potential gaps in biosecurity behaviours depending on the risk, type of trust, and control measure used. Beyond increased awareness and a shared concern, steps should be taken to build the profile of biosecurity as a collective action problem tackled by community endeavour. It is likely that trust and control are not mutually exclusive as some trust will exist alongside some control actions. Trust may take the place of controls over time, with the frequency or intensity of control measures decreasing as trust increases. For biosecurity in the UK plant trade interventions should focus on i) enhancing the ability of businesses to build biosecurity trust over time, and ii) tools to enable and normalise control actions.

Building trust between parties to work together for plant health is important to avoid individuals making a self-interested choice to subvert biosecurity practices for personal gain, yet we suggest that relying on goodwill trust in the presence of a pathogen such as Xf with low detectability could still result in a high-risk scenario (S2, Fig. 3), as a good working relationship could result in toleration of lower biosecurity standards. This apparent contradiction occurs because plant health is just one aspect of cooperation between businesses. Interventions for trust building should include creation of opportunities for open, ongoing cooperation on biosecurity between businesses and suppliers (as well as other stakeholders) where in addition to sharing best practices and plant health alerts, communities of businesses address biosecurity using strategies they can develop themselves. Repeated opportunity for face to face discussion has been shown to encourage cooperation and build trust (Ostrom et al. 1994). Our research focussed on specialist nurseries, garden centres, and landscapes professionals, and several interviewees noted suspicion of non-specialist plant retailers such as supermarkets. Building trust between various businesses who sell plants means sharing of different challenges faced, work done, and mistakes made to cross business category divides. A high-risk scenario was also identified in the case of a hard to detect pest or pathogen occurring when a business relies only on output control (S8, Fig. 3), illustrating the danger of a purely reactive management system in the presence of a hard to detect pest. Businesses should have access to tools for proactive pest management including knowledge of the right biosecurity questions to ask their suppliers. Businesses themselves should also be aware that they will be asked these questions by their customers and thus have the correct protocols and processes in place.

Interestingly, our survey indicated that a small number of suppliers (7%) were responsible for a proportion of lavender and cherry plants sold (over 75%). If this is due to a lack of choice in the market, then this could limit the choices available to a business should they not be content with their current supplier, forcing them to continue with a substandard supplier or stop providing their own customers with the product. It would also represent an opportunity for efficient points of intervention, targeting of these suppliers to better their biosecurity would be more efficient than targeting individual businesses. This aspect of our study warrants further investigation.

Lack of awareness can be a major barrier to UK plant businesses adopting greater biosecurity practices (Dunn and Marzano 2019). Thus, raising knowledge and awareness of practices through increased industry training in plant health and biosecurity is a logical first step to voluntarily raising biosecurity standards. Building shared concern is termed ‘social control’ in the risk-based view of trust, where it is identified as a foundational strategy to begin tackling either type of risk when trust and other controls fail. Social control for biosecurity aims for parties to agree that plant pests and pathogens are a risk, and that effort should be made to minimise that risk. Awareness raising alone cannot be expected to change behaviours, as even verified knowledge and concern will not always result in the desired behaviour change (Cook and Melo Zurita 2019). Due to their legitimacy, reach, and access to the most up-to-date information, industry bodies and government are well placed to conduct knowledge provision to businesses and individuals in the industry.

A role here can be played by industry-led certification schemes (e.g. Plant Healthy Certification Scheme in the UK, planthealthy.org.uk) which aim to encourage increased biosecurity of both individual businesses through certification against published standards, and of the wider sector through e.g. the potentially higher prices charged by certified businesses or through reputational damage of not taking part. Certification schemes enhance competence trust in the certified supplier, switching some of the trust which would be in the supplier to the certification scheme, allowing businesses to leave complicated and costly controls in the hands of the certification body rather than undertaking themselves.

## Conclusion

Trust is a significant factor for people in the live plant trade tackling the potential risks of sourcing decisions. Where trust isn’t present, control methods can be used to tackle risk instead. We have shown behaviours displayed in response to different types of trust and control, could result in varied biosecurity outcomes depending on the detectability of a pest or pathogen. Behavioural interventions designed to encourage greater biosecurity need to take into account the multiple types of risk people perceive, as those actors targeted for behaviour change may not see a need to act differently instead relying on their own trust and control systems to mitigate risks. Building a collective action response to biosecurity requires creation of opportunities which include forums for increasing instances of communication and tools to enable structured biosecurity discussion during such instances.

## References

[CR1] Bagavathiannan MV, Graham S, Ma Z, Barney JN, Coutts SR, Caicedo AL, De Clerck-Floate R, West NM, Blank L, Metcalf AL, Lacoste M, Moreno CR, Evans JA, Burke I, Beckie H (2019). Considering weed management as a social dilemma bridges individual and collective interests. Nature Plants.

[CR2] Brasier CM (2008). The biosecurity threat to the UK and global environment from international trade in plants. Plant Pathol.

[CR3] Chapman D, Purse BV, Roy HE, Bullock JM (2017). Global trade networks determine the distribution of invasive non-native species. Glob Ecol Biogeogr.

[CR4] European Commission (2018) Commission database of host plants found to be susceptible to *Xylella fastidiosa* in the union territory – update 11

[CR5] Cook BR, de Zurita MLM (2019). Fulfilling the promise of participation by not resuscitating the deficit model. Global Environ Change.

[CR6] Dandy N, Marzano M, Porth EF, Urquhart J, and Potter C (2017) “Who has a stake in ash dieback? A conceptual framework for the identification and categorisation of tree health stakeholders, pp. 15–26, in Dieback of European Ash (*Fraxinus spp*.): consequences and guidelines for sustainable management, edited by R. Vasaitis and R. Enderle. Swedish University pof Agricultural Sciences

[CR7] Das TK, Teng B-S (2001). Trust, control, and risk in strategic alliances. Organ Stud.

[CR8] Das TK, Teng BS (2004). The risk-based view of trust: a conceptual framework. J Bus Psychol.

[CR9] Defra (2020) Rapid pest risk analysis (PRA) for: *Xylella fastidiosa* summary and conclusions of the rapid PRA. Vol. 2020

[CR10] Drew J, Anderson N, Andow D (2010). Conundrums of a complex vector for invasive species control: a detailed examination of the horticultural industry. Biol Invasions.

[CR11] Dunn M and M Marzano (2019) Phyto-threats nurseries and garden centres survey summary report on attitudes towards biosecurity and accreditation

[CR12] Fulmer CA, Gelfand MJ (2011). At what level (and in whom) we trust: trust across multiple organizational levels. SSRN Electron J.

[CR13] Green S, Cooke DEL, Dunn M, Barwell L, Purse B, Chapman DS, Valatin G, Schlenzig A, Barbrook J, Pettitt T, Price C, Pérez-Sierra A, Frederickson-Matika D, Pritchard L, Thorpe P, Cock PJA, Randall E, Keillor B, Marzano M (2021). Phyto-threats: addressing threats to UK forests and woodlands from phytophthora; identifying risks of spread in trade and methods for mitigation. Forests.

[CR14] Hill L, Jones G, Atkinson N, Hector A, Hemery G, Brown N (2019). The £15 billion cost of ash dieback in Britain. Curr Biol.

[CR15] Hopkins DL, Purcell AH (2002). *Xylella fastidiosa*: cause of pierce’s disease of grapevine and other emergent diseases. Plant Dis.

[CR16] Hulme PE, Brundu G, Carboni M, Dehnen-Schmutz K, Dullinger S, Early R, Essl F, González-Moreno P, Groom QJ, Kueffer C, Kühn I, Maurel N, Novoa A, Pergl J, Pyšek P, Seebens H, Tanner R, Touza JM, van Kleunen M, Verbrugge LNH (2018). Integrating invasive species policies across ornamental horticulture supply chains to prevent plant invasions. J Appl Ecol.

[CR17] Jamaluddin F, Saibani N (2021). Systematic literature review of supply chain relationship approaches amongst business-to-business partners. Sustainability.

[CR18] Kleczkowski A, Hoyle A, McMenemy P (2019). One model to rule them all? modelling approaches across onehealth, for human, animal and plant epidemics. Philos Trans B Biol Sci.

[CR19] Kostis A, Näsholm MH (2019). Towards a research agenda on how, when and why trust and distrust matter to coopetition. J Trust Res.

[CR20] Lewicki RJ, Tomlinson EC, Gillespie N (2006). Models of interpersonal trust development: theoretical approaches, empirical evidence, and future directions. J Manag.

[CR21] Marzano M, Allen W, Haight RG, Holmes TP, Carina E, Keskitalo H, Lisa Langer ER, Shadbolt M, Urquhart J, Dandy N (2017). The role of the social sciences and economics in understanding and informing tree biosecurity policy and planning: a global summary and synthesis. Biol Invasions.

[CR22] Marzano M, Ambrose-Oji B, Hall C, Moseley D (2020). Pests in the city: managing public health risks and social values in response to oak processionary moth (*Thaumetopoea processionea*) in the United Kingdom. Forests.

[CR23] Marzano M, Dunn M, Green S (2021). Perceptions of biosecurity-based accreditation in the plant trade: a UK example. Forests.

[CR24] Mayer RC, Davis JH, David Schoorman F (1995). An integrative model of organizational trust. Acad Manag Rev.

[CR25] Mlaker KS, Gorenak I, Potocan V (2016). The influence of trust on collaborative relationships in supply chains. EM Ekonomie a Management.

[CR26] Occhibove F, Chapman DS, Mastin AJ, Parnell SSR, Agstner B, Mato-Amboage R, Jones G, Dunn M, Pollard CRJ, Robinson JS, Marzano M, Davies AL, White RM, Fearne A, White SM (2020). Eco-epidemiological uncertainties of emerging plant diseases: the challenge of predicting *Xylella fastidiosa* dynamics in novel environments. Phytopathology.

[CR27] Ostrom E, Gardner R, Walker J (1994). Rules, games and common-pool resources.

[CR28] Ostrom E, Burger J, Field CB, Norgaard RB, Policansky D (1999). Revisiting the commons: local lessons, global challenges. Science.

[CR29] Pautasso M, Jeger MJ (2014). Network epidemiology and plant trade networks. AoB Plants.

[CR30] Porth EF, Dandy N, Marzano M (2015). ‘My garden is the one with no trees:’ residential lived experiences of the 2012 Asian Longhorn Beetle eradication programme in Kent, England. Hum Ecol.

[CR31] Rapicavoli J, Ingel B, Blanco-Ulate B, Cantu D, Roper C (2018). Xylella fastidiosa: an examination of a re-emerging plant pathogen. Mol Plant Pathol.

[CR500] R Core Team (2020). R: A language and environment for statistical computing. R Foundation for Statistical Computing, Vienna. https://www.R-project.org/

[CR32] Rinehart LM, Eckert JA, Handfield RB, Page TJ, Atkin T (2004). An assessment of supplier: customer relationships. J Bus Logist.

[CR33] Saponari M, Giampetruzzi A, Loconsole G, Boscia D, Saldarelli P (2019). Xylella Fastidiosa in olive in Apulia: where we stand. Phytopathology.

[CR34] Schoorman FD, Mayer RC, Davis JH (2007). An integrative model of organizational trust: past, present, and future. Acad Manag Rev.

[CR35] Scortichini M (2020). The multi-millennial olive agroecosystem of Salento (Apulia, Italy) threatened by *Xylella Fastidiosa* Subsp. Pauca: a working possibility of restoration. Sustainability.

[CR36] Spence N, Hill L, Morris J (2020). How the global threat of pests and diseases impacts plants, people, and the planet. Plants People Planet.

[CR37] Stern MJ, Coleman KJ (2015). The multidimensionality of trust: applications in collaborative natural resource management. Soc Nat Resour.

